# Haemoptysis treated by bronchial artery embolisation in severe acute respiratory syndrome coronavirus 2: case report

**DOI:** 10.1186/s42155-020-00154-x

**Published:** 2020-09-05

**Authors:** Salvatore Murgo, Olivier Lheureux, Fabio Taccone, Michael Vouche, Jafar Golzarian

**Affiliations:** 1 Erasme Hospital, ULB, route de Lennik, 808, 1070 Brussels, Belgium; 2 University of Minnesota Medical Center, Minneapolis, USA

**Keywords:** SARS-CoV-2, COVID-19, Haemoptysis, Embolisation, Bronchial artery

## Abstract

**Background:**

We found no data in the literature on the embolization of the bronchial arteries in the context of hemoptysis associated with severe acute respiratory syndrome coronavirus 2. We therefore decided to share this experience.

**Case presentation:**

A 62-year-old patient with no significant medical history was admitted with acute respiratory distress. Chest computed tomography showed diffuse bilateral ground-glass opacities with limited consolidations. Diagnostic tests confirmed severe acute respiratory syndrome coronavirus 2 infection. The severity of respiratory failure required the implantation of veno-venous extracorporeal membrane oxygenation. The patient developed severe haemoptysis, which was successfully treated by bronchial artery embolisation.

**Conclusions:**

In the case of coronavirus-19 pneumonia, our experience suggests that the treatment of severe haemoptysis by bronchial artery embolisation is feasible and effective. The survival benefit should be assessed in the future.

## Introduction

At the end of December 2019, severe acute respiratory syndrome coronavirus 2 (SARS-CoV-2) emerged in Hubei Province, China, leading to the global outbreak of coronavirus disease 2019 (COVID-19) (Zhu et al. 2020). Studies have shown that the virus probably originated in a seafood market in Wuhan, but specific animal associations have not yet been confirmed (Zhu et al. 2020). With this new virus, we are confronted with new logistical, diagnostic, therapeutic, and human challenges, particularly since it has a surprising diversity of clinical presentations that we are still discovering.

Faced with a case of severe haemoptysis related to COVID-19 pneumonia, we took an emergency therapeutic decision. However, we found no data in the medical literature to support our approach. We therefore decided to share this experience.

## Case report

**Fig. 1:**
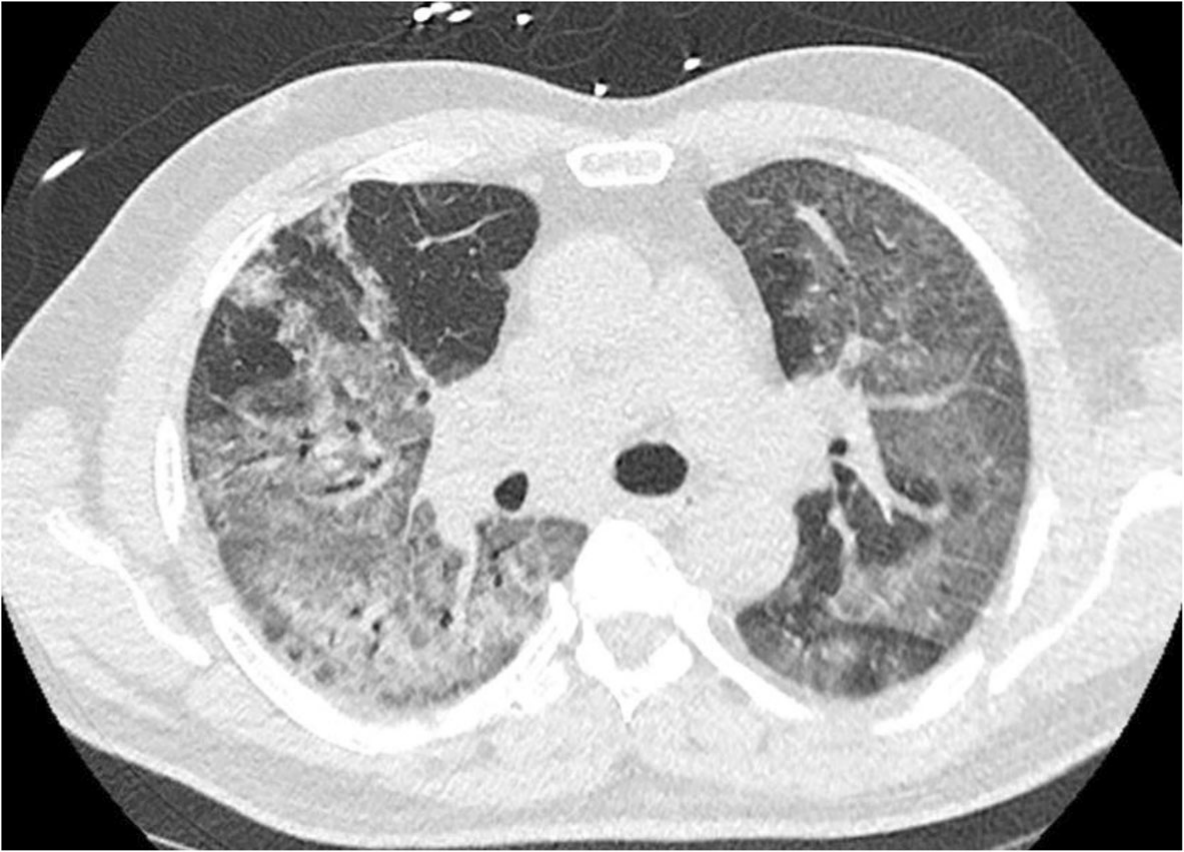
Chest computed tomography showing diffuse bilateral ground-glass opacities with limited consolidations

On 14 March 2020, a 62-year-old male patient with no significant medical history was admitted to intensive care for acute respiratory distress associated with cough, fever, and oxygen desaturation. Chest computed tomography (CT) was performed on admission, revealing diffuse bilateral ground-glass opacities with limited consolidations (Fig. 1). Biological analysis showed an important inflammatory syndrome and kidney failure. Baseline laboratory values showed a slightly prolonged activated partial thromboplastin time (APTT) and increased D-dimer level which have both been reported in COVID-19 patients and associated with poor prognosis (Tang et al. 2020). We observed normal prothrombin time (PT), fibrinogen levels, and no thrombocytopenia. On the day of admission, the patient was intubated with ventilatory strategies similar to those applied in the case of severe acute respiratory distress syndrome such as high positive end expiratory pressure (PEEP) and prone positioning. Empirical antibiotic treatment with cefuroxime was administered.

Polymerase chain reaction (PCR) on a nasal swab confirmed COVID-19 diagnosis, and a bronchoalveolar lavage showed no bacterial infection. Following local guidelines, the patient was treated with hydroxychloroquine for 10 days. The patient developed renal failure that required dialysis. On day 2, refractory hypoxemia required the implantation of veno-venous extracorporeal membrane oxygenation (ECMO). Anticoagulant therapy with unfractionated heparin (UNFH) was immediately started after implantation as recommended by the Extracorporeal Life Support Organisation guidelines. According to local protocol, APTT was used to monitor and adjust UNFH therapy.

On day 9, the patient developed life-threatening haemoptysis, and the chest x-ray showed increased condensation, particularly in the right lung. Several bronchoscopies were required to remove the recurring clots, particularly in the right main bronchus. Diffuse bleeding was seen on both sides. No heparin overdose was detected. Heparin treatment was stopped for 24 h. Viscoelastic coagulation tests (ROTEM®) were normal, while there was no effect of aerosolised tranexamic acid or intravenous desmopressin in the case of acquired Von Willebrand disease associated with ECMO therapy.

Faced with recurrent haemoptysis, bronchial artery embolisation was considered. We found no data in the medical literature on COVID 19 and bronchial artery embolization. We contacted several colleagues in France and the United States, but none had encountered this situation. Faced with this massive hemoptysis and without embolization, the patient’s death seemed inevitable.

**Fig. 2:**
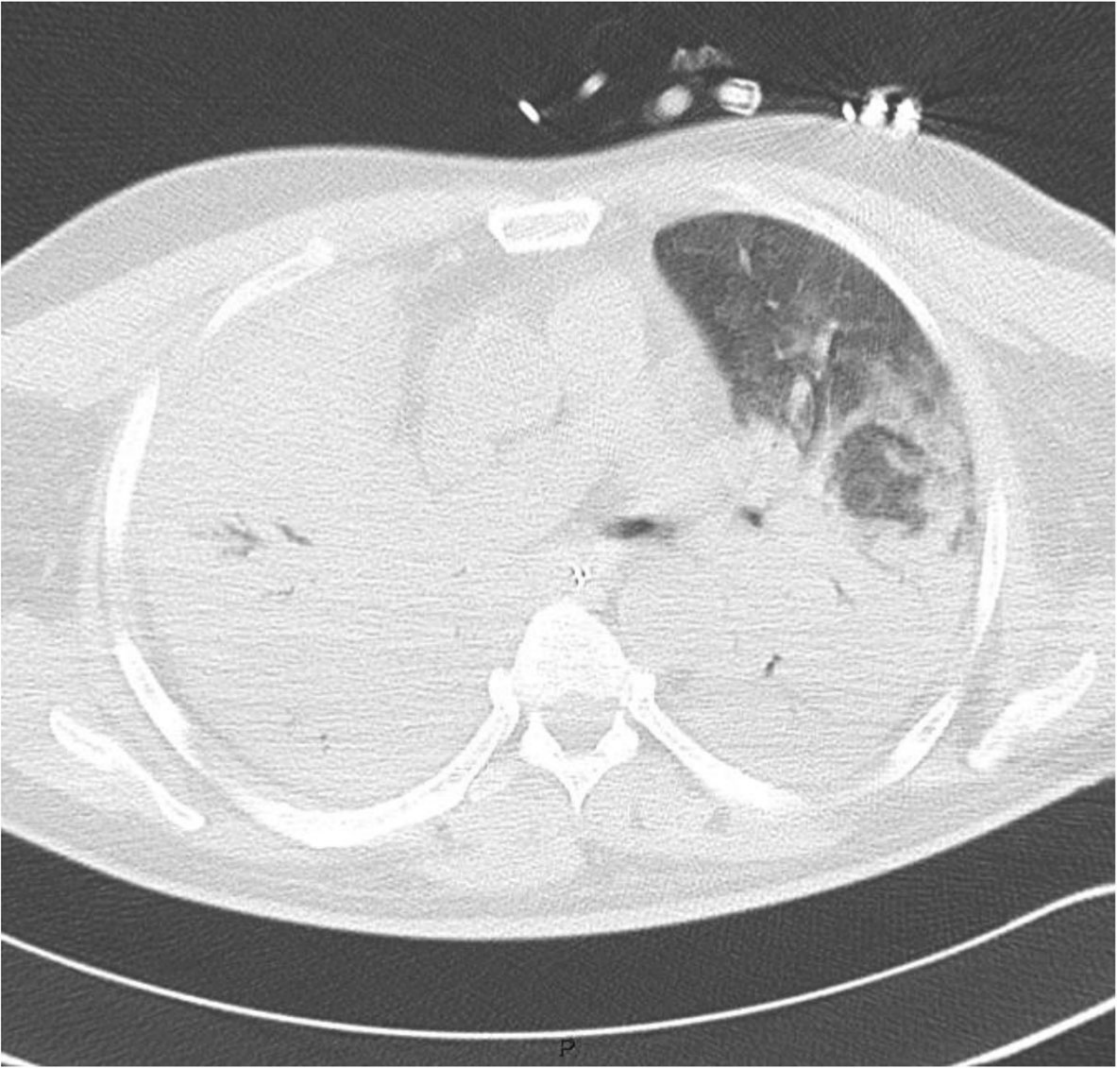
Chest computed tomography angiography without vascular abnormalities. A complete condensation of the right lung and three-quarter condensation of the left lung may be observed

Chest CT angiography (CTA) was performed prior to embolisation. It showed no pulmonary embolism or vascular abnormalities but the complete condensation of the right lung and three-quarter condensation of the left lung (Fig. 2).

**Fig. 3:**
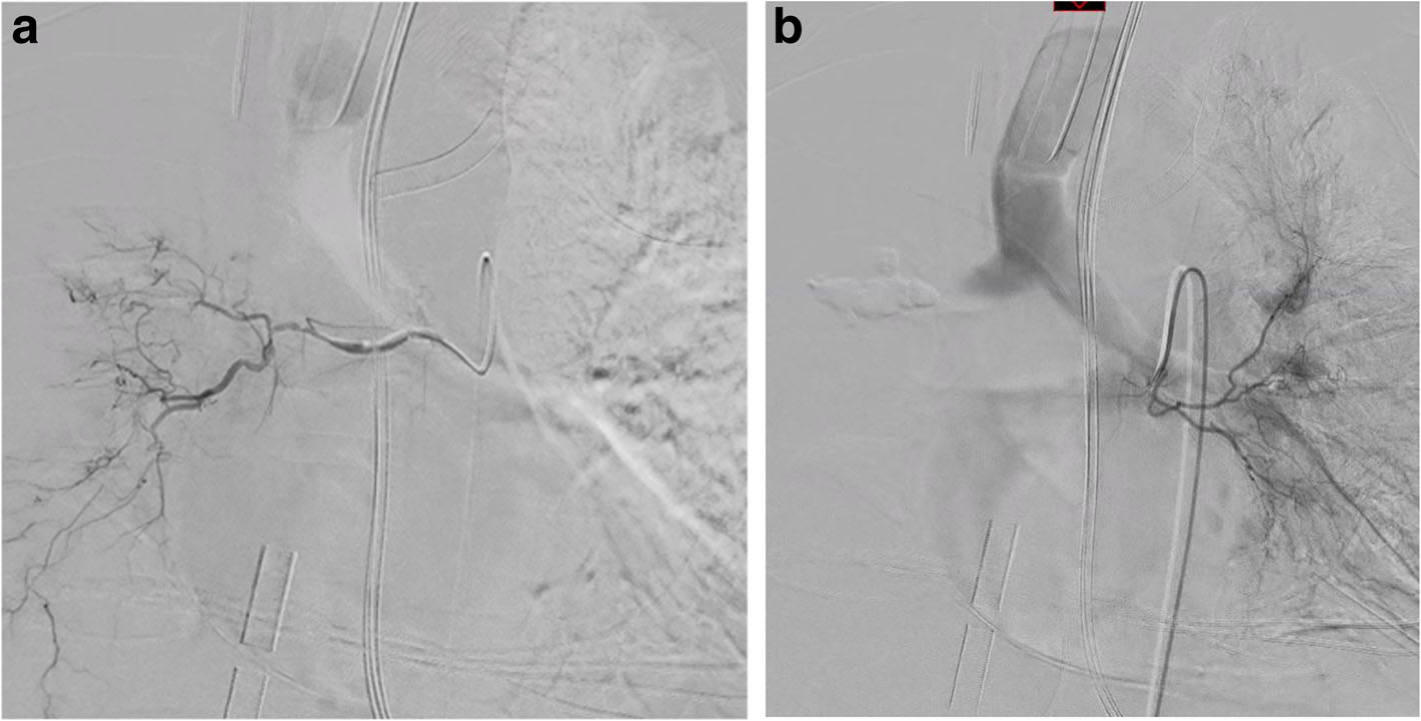
Arteriography showing normal right (**a**) and left (**b**) bronchial arteries

Through a right arterial femoral access with a 4 French (F) introducer sheath, we performed selective bronchial arteriographies using a 4F Sidewinder 1 Catheter (Cordis) and 2.7F microcatheter (Progreat, Terumo), which showed the normal appearance and calibre of the right and left bronchial arteries (Fig. 3) without active bleeding. Despite the advanced lung damage, we decided to proceed with the embolisation to control the haemoptysis. Complete bilateral embolisation was performed using calibrated particles (Embozene® 400 μm, Varian Medical Systems).

After the embolisation procedure, no recurrence of haemoptysis occurred, even after resuming anticoagulant treatment with UNFH. The chest X-ray revealed a slight improvement in the ventilation of the right lung. Unfortunately, the patient died from non-haemorrhagic shock3 days post-embolisation.

## Conclusions

The full spectrum of COVID-19 disease is still emerging, but the most common signs and symptoms at onset are fever, dry cough, myalgia, anosmia and ageusia, fatigue, and dyspnoea (Guan et al. 2020; Lapostolle et al. 2020).

Pulmonary bleeding seems to be an atypical manifestation of SARS-CoV-2 infection, as COVID-19-associated haemoptysis has rarely been reported in the literature (Lapostolle et al. 2020). In a selected Chinese cohort, haemoptysis was present in 0.9% of 1099 COVID-19 patients (2.3% in severe cases) (Guan et al. 2020). Recently, Lapostolle et al. reported a 3% haemoptysis rate in a large cohort of confirmed COVID-19 patients managed in an outpatient setting (Lapostolle et al. 2020).

Very few major haemorrhagic complications have been described in hospitalised COVID-19 patients, with only a few cases of spontaneous abdominal internal bleeding and none at the bronchial level (Conti et al. 2020). Minor or occult bleeding was reported, especially due to SARS-CoV-2 associated digestive tract lesions (Lin et al. 2020).

A hallmark of severe COVID-19 is altered coagulation with a predominantly pro-thrombotic status and a high risk of thromboembolic events (Helms et al. 2020). The initial coagulopathy of COVID-19 presents a prominent elevation of D-dimer and fibrin/fibrinogen degradation products, while abnormalities in PT, APTT, and platelet counts are relatively uncommon in initial presentations (Connors and Levy 2020). In some centres, venous and pulmonary thromboembolic complications are found in one-third of critically ill patients, leading to prophylactic doses higher than the thrombosis prophylaxis recommendation even in the absence of randomised evidence.

The lungs are the target organ for SARS-CoV-2, and progressive respiratory failure is the primary cause of death in COVID-19 patients. Pathological examination of the lungs of deceased COVID-19 patients showed diffuse alveolar damage associated with both thrombotic and haemorrhagic lesions: severe endothelial injury, alveolar capillary microthrombi, and increased angiogenesis (Ackermann et al. 2020).

In this context, we hypothesised that the life-threatening haemoptysis presented by our patient was consecutive to severe lung damage induced by SARS-CoV-2 combined with the anticoagulant effect of UNFH therapy, resulting in peripheral bronchial artery damage. In such cases, bronchial artery embolisation could be indicated.

Faced with haemoptysis, chest CTA is recommended to specify the nature, extent, and topography of pulmonary lesions. CTA allows the evaluation of the bronchial artery anatomy and the exploration of pulmonary arteries, which are a less common cause of haemoptysis. A recent study showed that preprocedural CTA helps detect culprit ectopic bronchial arteries and non-bronchial systemic arteries originating from the subclavian and internal mammary arteries during bronchial artery embolisation (Li et al. 2019). Li et al. observe that chest CTA improve the haemoptysis-free early survival rate in patients with haemoptysis (Li et al. 2019).

At the time of writing, this is the first reported case of bronchial artery embolisation used for COVID-19-related haemoptysis. Our experience suggests that the embolisation of bronchial arteries is feasible and may help control bleeding in cases of COVID-19 pneumonia associated with life-threatening haemoptysis. However, the impact on survival remains uncertain. The risk incurred by medical and paramedical staff is an issue that must also be taken into account. We hope that other authors will report their similar experiences in order to provide answers to these questions.

## Data Availability

Available in the computer files of our hospital. Data are however available from the authors upon reasonable request.

## Abbreviations

SARS-CoV-2: Severe acute respiratory syndrome coronavirus 2
COVID 19: Coronavirus disease 2019
CT: Computed tomography
APTT: Activated partial thromboplastin time
PT: Prothrombin time
PEEP: High positive end expiratory pressure
PCR: Polymerase chain reaction
ECMO: Extracorporeal membrane oxygenation
UNFH: Unfractionated heparin
CTA: CT angiography
F: French
